# FDG PET Parkinson’s disease-related pattern as a biomarker for clinical trials in early stage disease

**DOI:** 10.1016/j.nicl.2018.08.006

**Published:** 2018-08-10

**Authors:** Dawn C. Matthews, Hedva Lerman, Ana Lukic, Randolph D. Andrews, Anat Mirelman, Miles N. Wernick, Nir Giladi, Stephen C. Strother, Karleyton C. Evans, Jesse M. Cedarbaum, Einat Even-Sapir

**Affiliations:** a ADM Diagnostics Inc., USA; b Tel Aviv Sourasky Medical Center, Tel Aviv, Israel; c Biogen, Cambridge, MA, USA; d Medical Imaging Research Center, Illinois Institute of Technology, Chicago, IL, USA; e Rotman Research Institute, Baycrest, Toronto, Ontario, CA, Canada; f Sackler Faculty of Medicine, Tel Aviv University, Tel Aviv, Israel; g Sagol School of Neuroscience, Tel Aviv University, Tel Aviv, Israel

**Keywords:** Parkinson, FDG PET, PDRP, Classifier, Biomarker

## Abstract

**Background:**

The development of therapeutic interventions for Parkinson disease (PD) is challenged by disease complexity and subjectivity of symptom evaluation. A Parkinson's Disease Related Pattern (PDRP) of glucose metabolism via fluorodeoxyglucose positron emission tomography (FDG-PET) has been reported to correlate with motor symptom scores and may aid the detection of disease-modifying therapeutic effects.

**Objectives:**

We sought to independently evaluate the potential utility of the PDRP as a biomarker for clinical trials of early-stage PD.

**Methods:**

Two machine learning approaches (Scaled Subprofile Model (SSM) and NPAIRS with Canonical Variates Analysis) were performed on FDG-PET scans from 17 healthy controls (HC) and 23 PD patients. The approaches were compared regarding discrimination of HC from PD and relationship to motor symptoms.

**Results:**

Both classifiers discriminated HC from PD (p < 0.01, p < 0.03), and classifier scores for age- and gender- matched HC and PD correlated with Hoehn & Yahr stage (R^2^ = 0.24, p < 0.015) and UPDRS (R^2^ = 0.23, p < 0.018). Metabolic patterns were highly similar, with hypometabolism in parieto-occipital and prefrontal regions and hypermetabolism in cerebellum, pons, thalamus, paracentral gyrus, and lentiform nucleus relative to whole brain, consistent with the PDRP. An additional classifier was developed using only PD subjects, resulting in scores that correlated with UPDRS (R^2^ = 0.25, p < 0.02) and Hoehn & Yahr stage (R^2^ = 0.16, p < 0.06).

**Conclusions:**

Two independent analyses performed in a cohort of mild PD patients replicated key features of the PDRP, confirming that FDG-PET and multivariate classification can provide an objective, sensitive biomarker of disease stage with the potential to detect treatment effects on PD progression.

## Highlights

• The Parkinson's disease-related pattern (PDRP) of glucose metabolic effects is demonstrated in an independent cohort of early stage PD patients.
• The PDRP pattern of metabolic changes is robust to variations in image processing and choice of classification model.
• Age-related metabolic changes show partial overlap with the PDRP, suggesting that age-adjustment is an important consideration.
• The PDRP correlates with motor function as defined by Hoehn & Yahr stage and UPDRS score.
• An additional data driven metabolic classifier highlights pattern aspects associated with early stage motor decline.

## Graphical abstract

**Figure:**
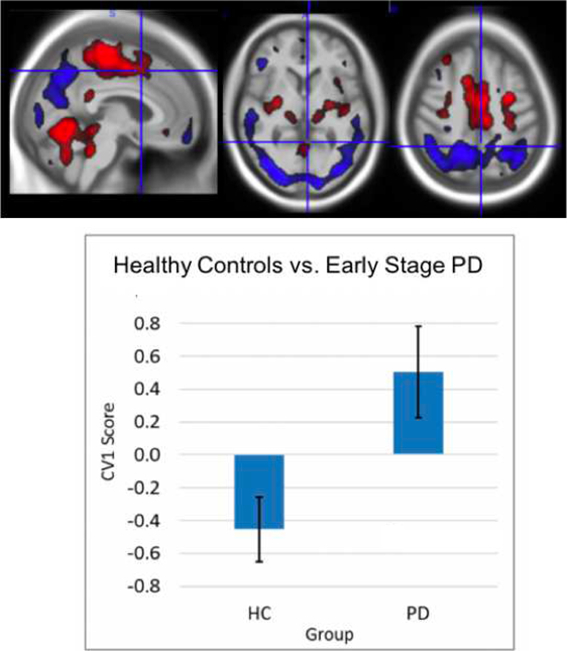

## 1 Introduction

The complex effects of Parkinson disease (PD), which include multi-faceted motor symptoms, cognitive effects, and non-motor symptoms, pose challenges in measuring treatment effect upon disease progression. Although the Unified Parkinson's Disease Rating Scale III (UPDRS-III) (Goetz et al., 2008) provides a broadly used measure of motor deterioration, more sensitive, objective measures of decline are needed (Heldman et al., 2011). Several published reports have explored the potential of glucose metabolism measurement using 2-deoxy-floroglucose positron emission tomography (FDG-PET) as a biomarker of PD progression for clinical trials. The present study sought to determine whether the Parkinson's Disease Related Pattern (PDRP) (Eidelberg et al., 1994; Ma et al., 2007) observed in previously published FDG-PET studies could be independently replicated in early stage PD subjects.

Regional cerebral glucose metabolism, the primary energy source for neuronal activity (Dienel and Hertz, 2001), reflects changes in neuronal function due to disease, therapeutic intervention, and cognitive or physical activity. Using FDG-PET and multivariate machine learning techniques, a Parkinson's Disease Related Pattern (PDRP) has been identified, characterized by hypometabolism in parieto-occipital and premotor cortices concomitant with metabolic preservation or hypermetabolism in cerebellum, pons, thalamus and lentiform nucleus (Eidelberg et al., 1994; Ma et al., 2007; Tomše et al., 2017a). The pattern is consistent with regional findings in other studies in PD patients (Kuhl et al., 1984; Bohnen et al., 1999; Bohnen et al., 2011). The PDRP has been demonstrated to correlate with cross-sectional disease severity and UPDRS motor scores (Eidelberg et al., 1994; Ma et al., 2007; Huang et al., 2013; Wu et al., 2013), longitudinal disease progression (Huang et al., 2007), and therapeutic response (Feigin et al., 2001; Feigin et al., 2007). Subjects with Parkinson's symptoms but without dopaminergic deficit have been found not to express the PDRP in contrast to PD patients with dopaminergic deficit (Eckert et al., 2007). It has also been applied to evaluate scans in comparison to other similarly derived disease related patterns to discriminate between idiopathic PD, multiple system atrophy, and progressive supranuclear palsy (Teune et al., 2013; Mudali et al., 2015; Tripathi et al., 2016). The pattern has been demonstrated using several different data sets from a variety of countries and ethnic populations (Eidelberg et al., 1994; Ma et al., 2007; Teune et al., 2013; Wu et al., 2013; Tomše et al., 2017a), PET scanners (Moeller et al., 1999), and reconstruction algorithms (Tomše et al., 2017b). The published PDRP studies to-date share a common analytic approach and all but one of these studies share at least one common author/co-author despite evaluating several unique data sets. The PDRP has yet to be confirmed in a unique data set by an independent group of authors using different analytic techniques.

The primary objective of our work was to confirm that the PDRP could be detected in a mild PD patient population using two different machine learning approaches by an independent research group. The second objective was to evaluate the relationship between metabolic pattern expression and motor scores. The first machine learning approach used the Standard Subscale Profile (SSP) Method (Moeller and Strother, 1991) that has identified the PDRP in multiple PD populations as described above. The second approach used a different image pre-processing sequence and applied Canonical Variates Analysis within the Non-parametric Activation and Influence Reproducibility Resampling (NPAIRS) framework (Strother et al., 2002; Strother et al., 2010). NPAIRS was previously developed to address the common problem of overfitting in machine learning, and uses intensive, iterative split half resampling of the data set to generate metrics of reproducibility (correlation between models derived for each half of the data set) and prediction (correct classification of test half based upon training half) for selection of model parameters that optimize classifier stability and generalizability.

## 2 Methods

### 2.1 Study participants

FDG-PET data were acquired in 17 Healthy Controls (HC) and 25 PD patients at the Tel-Aviv Sourasky Medical Center, between the years 2011 and 2015. The data were collected under Institutional Review Board approval with informed consent by participants. Subjects were characterized (in the on medication state) with the Hoehn & Yahr (H & Y), Unified Parkinson's Disease Rating Scale (UPDRS) and Montreal Cognitive Assessment (MoCA) scales (Nasreddine et al., 2005). PD patients were of both sporadic and autosomal dominant genetic origin (G2019S-LRRK2 mutation).

### 2.2 Image acquistion

All FDG-PET scans were acquired in the morning at approximately 11 am. Patients were asked to stop anti-Parkinson medication on the day of the PET scan. Blood glucose levels were measured prior to the injection of fluorodeoxyglucose and verified to be <160 mg/ml in all study patients. 3D brain PET acquisition was performed using a GE Discovery 690 PET-CT scanner 30 min after the IV administration of 5-7 mCi (185-259 MBq) fluorodeoxyglucose over a period of 15 min. Subjects remained with eyes open at rest in a dimly lit room during the tracer uptake period. Images were attenuation corrected using a CT scan of 120 kV and automated mA, and reconstructed using a 2.5 mm slice thickness and the standard PET-CT reconstruction algorithms on the GE 690 Discovery system.

### 2.3 Data quality control and processing

Reconstructed images, produced in static form (a single timeframe), were visually inspected for anatomical completeness and to ensure suitability for processing. Images were spatially transformed to a common PET template using a PET to PET transformation in the Statistical Parametric Mapping software SPM8 (http://www.fil.ion.ucl.ac.uk/spm/software/spm8). Images were smoothed for group analysis using an 8 mm Gaussian filter kernel, full width at half maximum.

### 2.4 Multivariate pattern analysis

As noted above, the classifier development was implemented using two approaches: (1) Scaled Subprofile Model (SSM) analysis (Moeller and Strother, 1991; Eidelberg et al., 1994; Spetsieris and Eidelberg, 2011) and (2) Canonical Variates Analysis (CVA, a form of linear discriminant analysis) as implemented within NPAIRS (Strother et al., 2002; Strother et al., 2010). The two approaches are similar in their use of supervised learning with defined training classes, Principal Component Analysis (PCA) to identify differentiating features between groups, and linear discriminant analysis to combine selected Principal Components (PCs) into a final model (set of patterns). The approaches differed in the intensity normalization methods applied to the images that were input to the machine learning model (SSM vs. z-scoring), the algorithm applied to mathematically combine selected Principal Components, and the method of using iterative data resampling to create a consensus classifier (NPAIRS).

When applying the SSM approach, the logarithm of each voxel value was calculated for each scan, the mean of the scan volume was removed from the voxels within the scan, and the mean of each voxel across all scans was additionally removed from that voxel (Moeller and Strother, 1991; Spetsieris and Eidelberg, 2011). The pre-processed scans, designated into training groups, were input to PCA, which identified uncorrelated patterns discriminating groups. Principal Components (PCs) were chosen and combined using logistic regression. To verify agreement between our implementation of SSM/PCA and that used in other published studies, analyses were performed using both published software (http://www.fil.ion.ucl.ac.uk/spm/ext) (Peng et al., 2014) and in-house software (www.admdx.com). In the initial analysis, the number of PCs was selected to include those that accounted for >5% of variance.

In the NPAIRS CVA approach, PCA was applied for feature reduction to images that had been intensity normalized by z-scoring to whole brain, grouped into pre-specified training classes (defined as in the SSM approach), and mean centered. CVA was then performed to combine selected PCs into a set of intensity patterns that best accounted for variance across the classes. For comparison to SSM, the same number of PCs was selected as the basis for CVA as was selected for the SSM approach. A consensus pattern was produced based upon the multiple split-half resampling iterations. A numeric canonical variates score (CV score) was calculated for each scan, reflecting the degree to which the scan expressed the associated pattern of intensities.

For each classification model, results were tested using a Leave-One-Out algorithm that allows testing of each data point without incorporating that data into model development. For the set of N subjects, the classifier was developed N times, each time leaving out a different subject and using N-1 subjects to develop the model, with PC selection based upon model performance, against which the Nth subject's scan was scored. This produced a data set consisting entirely of test scores, using the limited available number of subjects. As a separate, qualitative assessment, the resulting patterns were compared to those that have been published using completely different subject cohorts, and tested using independent subjects.

The same images, smoothing, and whole brain mask were used for both analysis approaches. Image processing and classifier development and testing (other than that performed using the on-line SSM software for verification) were performed using PipelineMAX™ software (www.admdx.com), which enables configuration of image processing pipelines, implements NPAIRS CVA and other classification approaches, produces metrics enabling comparison of classifier performance, performs nested Leave-N-Out testing, and creates a complete audit trail. Fig. 1 in the supplementary material summarizes the SSM and NPAIRS CVA approaches employed.

**Fig. 1:**
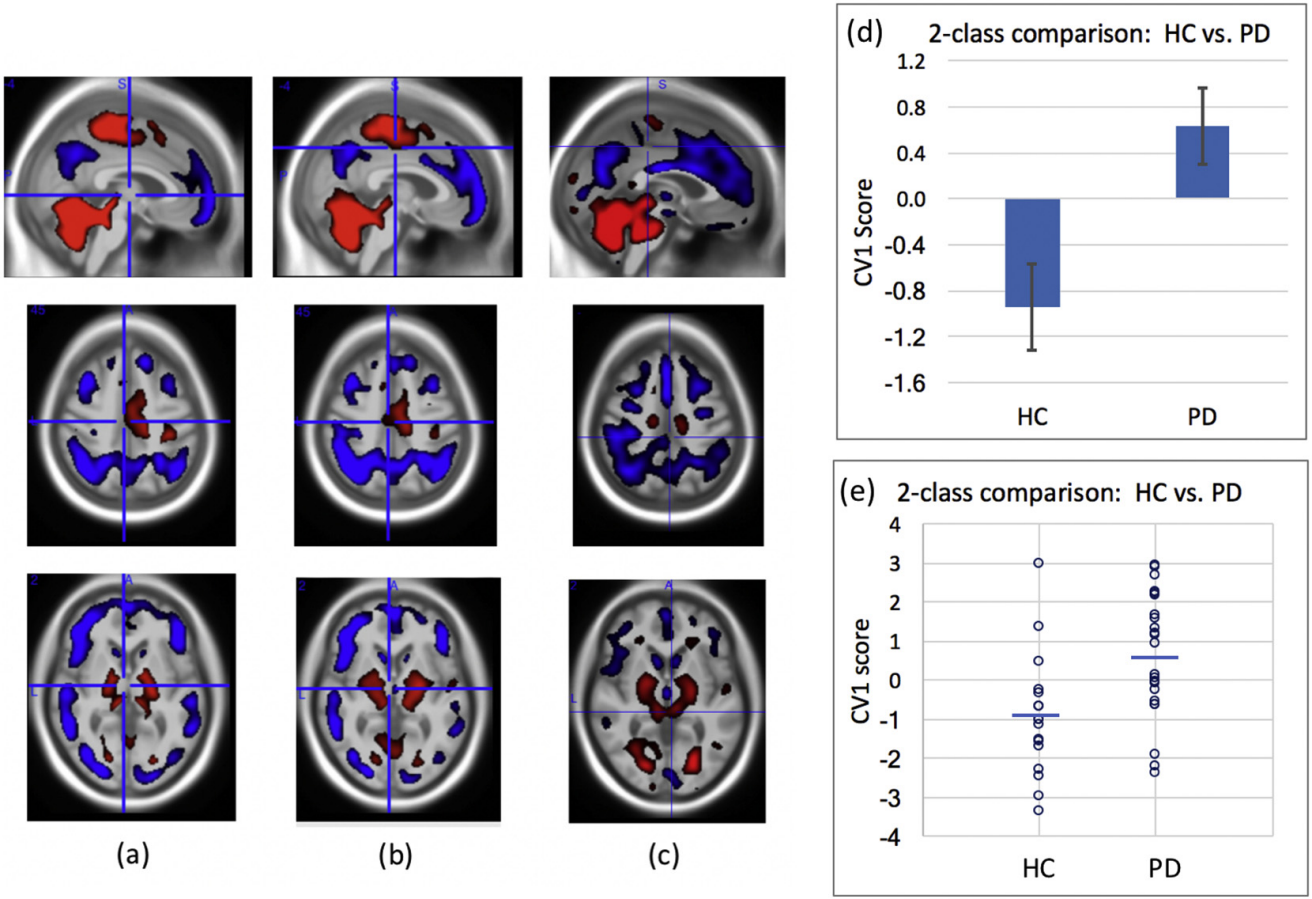
(a) SSM derived training pattern, (b) NPAIRS CVA derived consensus training pattern, (c) Leave-One-Out consensus test pattern from NPAIRS CVA, and comparison of Leave-One-Out test CV1 mean (d) and individual (e) scores for HC vs. PD subjects. Higher CV1 scores reflect greater magnitude of expression of the PDRP pattern. Red regions represent areas of increased metabolism, blue regions represent areas of decreased metabolism (relative to whole brain metabolism). Error bars show standard error of the mean. (For interpretation of the references to colour in this figure legend, the reader is referred to the web version of this article.)

### 2.5 Classifier design

*Healthy controls compared to Parkinson's disease.* The first analysis was based upon a comparison of the 17 HC and 23 PD subjects who passed QC. Two subjects were excluded from analysis: one due to poor spatial warping and a second because their UPDRS score was in the normal range despite a clinical diagnosis of PD. Analyses were first performed without further age-matching, in order to make use of the subjects available. Initial analyses were also performed without introducing age as a covariate or pattern projection into the model development, to systematically examine potential effects. Age influence was then examined by evaluating classifier scores for correlation with age within the HC group and separately within the PD group. Using HC subgroups stratified by age (10 HC age < 50 years of age vs. 7 HC subjects ≥50 years), an age-focused classifier was developed to compare potential overlap with the PD pattern. The pre-specified age partition cut-off of (age 50) was selected in order to balance the number of subjects and age range across groups. An additional classifier comparing PD subjects to healthy controls was then developed using a subset of age and gender matched groups (12 HC, 12 PD).

## 3 Motor symptom severity within Parkinson's subjects

Given that the PD cohort in the present study were characterized on the mild end of the PD spectrum, analyses of symptom severity were focused on discrimination between Hoehn & Yahr Stages I and II and on correlation to motor scores. Accordingly, a classifier was developed consisting of two classes: H&Y Stage I (N = 13) and the combination of H&Y Stage II (N = 9) and III (N = 1). Age and MoCA scores were balanced across the two classes. Resulting classifier scores from Leave-One-Out testing were examined in relationship to H&Y score and to UPDRS scores.

## 4 Individual subject examination

*Z*-score images were generated for individual subjects, using the HC set as the reference set, and dividing the reference set into a younger and older reference group to minimize age-related impact. The images of PD subjects were examined to explore whether H&Y stage I subjects might exhibit asymmetry or other differences relative to stage II subjects.

### 4.1 Statistical analyses

For each classifier, scores (CV or SSM) were compared across diagnostic groups using descriptive statistics. Effect sizes (E.S.) were calculated using the software tool G*Power (Faul et al., 2007; Faul et al., 2009). Correlation analyses were performed using Pearson's R (or Spearman's r when group sizes were 12 or less), and between-group comparisons and translation of r-values to *p*-values were performed via two-tailed *t*-test (p < 0.05).

## 5 Results

### 5.1 Subjects

Demographic and clinical characteristics of the subjects are shown in Table 1. At time of scan (in the on medication state), fifteen subjects were clinically diagnosed as Hoehn & Yahr (H&Y) Stage I, 9 as H&Y Stage II, and 1 as H&Y Stage III (mean = 1.44 ± 0.58 S.D.). Age ranged from 29 to 82 years. The HC group was somewhat younger than the PD group (HC vs. all PD, p < 0.01; HC vs. PD H&Y stage 1 p < 0.002), although age varied widely within each group. There was no significant age difference between PD subjects of H&Y Stage I vs. Stages II and III.

**Table 1:** Subject characteristics (on medication).

Group	N	Age mean (S.D.) range	Sex (%F)	Disease duration (years)	UPDRS motor	H&Y	MoCA
Controls (HC)	17	47.6 (11.8) 32 to 73	47%	n/a	1.3 (1.7)	0.0 (0.0)	27.4 (2.1)
PD (all)	25	59.0 (13.0) 29 to 82	35%	1.6 (1.3)	15.5 (7.4)	1.4 (0.6)	25.6 (2.0)
H&Y Stage I	15	61.4 (10.0) 42 to 82	40%	1.4 (1.1)	10.7 (3.1)	1.0 (0.0)	25.7 (1.7)
H&Y Stage II	9	57.8 (16.4) 29 to 80	33%	1.9 (1.7)	21.8 (4.9)	2.0 (0.0)	26.1 (2.2)
H&Y Stage III	1	37	0%	1.0	32 (n/a)	3.0 (0.0)	26 (n/a)

Values are mean, (S.D.) and range where applicable

N = number of subjects; S.D. = standard deviation; F = female; UPDRS = Unified Parkinson's Disease Rating Scale; H&Y = Hoehn & Yahr; MoCA = Montreal Cognitive Assessment; HC = Healthy Controls; PD = Parkinson Disease.

UPDRS scores correlated with and differed between H&Y stages; Stage I = 10.7 ± 3.1, Stage II = 21.8 ± 4.9, and Stage III = 32 (one subject). The HC group had higher MoCA scores than the PD group (p < 0.01). MoCA scores were not significantly different between PD subjects of H&Y Stage I vs. Stages II and III. Mean scores for each PD H&Y stage group were similar to the cutoff of 26 to discriminate normal cognition from impairment identified in the literature (Nasreddine et al., 2005; Hoops et al., 2009; Dalrymple-Alford et al., 2010; Kandiah et al., 2014) and higher (more normal) than a recently proposed revised cutoff of 23 (Carson et al., 2018). Three HC and 12 PD had scores below 26, with a minimum score of 23.

### 5.2 2-class analysis of HC and PD

Fig. 1 presents the training patterns produced by SSM (2a) and NPAIRS CVA (2b), and the Leave-One-Out consensus pattern and numeric group means (and SEM) produced by NPAIRS CVA (2c) for the two-class comparison of 17 HC and 23 PD subjects (13 H&Y Stage I, 9 Stage II, 1 Stage III). Patterns produced by SSM and NPAIRS CVA were both characterized by decreased glucose metabolism in posterior parietal, occipital, and premotor (and medial frontal) cortices, and increased glucose metabolism in cerebellum, pons, thalamus, lentiform nucleus (globis pallidus, putamen), and paracentral gyrus (all relative to whole brain). Premotor cortex hypometabolism extended into the anterior cingulate cortex. In Leave-One-Out testing, the patterns discriminated HC from PD (SSM p < 0.016, E.S. = 0.71; NPAIRS CVA p < 0.003, E.S. = 0.99).

### 5.3 Age effects

The CV1 scores of both two-class classifiers derived using 17 HC and 23 PD subjects correlated with age, both within the PD group (NPAIRS classifier R-squared = 0.30, p < 0.007) and within the HC group (NPAIRS classifier R-squared = 0.36, p < 0.011).

**Fig. 2:**
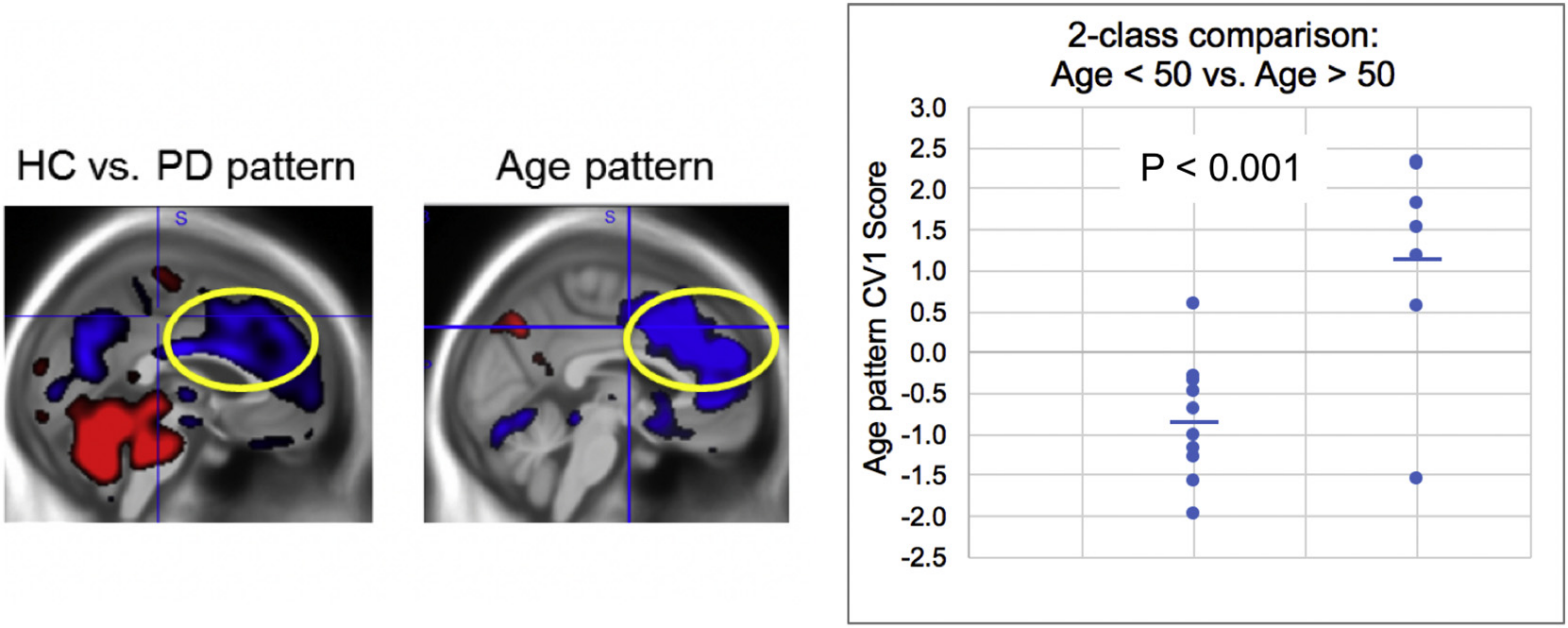
Age-related pattern identified by NPAIRS CVA in the comparison of HC subjects <50 and ≥ 50 years of age (graph and right image). The yellow oval focuses on the overlap between the age pattern (right) and the pattern comparing HC and PD subjects (left), where the PD subjects were somewhat older than HC on average. Results are split-half test results (consensus pattern generated using test results of many iterative data splits). Horizontal bars represent group means.

The 2-class NPAIRS comparison of 10 HC age < 50 to 7 HC age ≥ 50 from the data set produced a pattern of hypometabolism and hypermetabolism relative to whole brain that discriminated the two age groups (p < 0.001). As shown in Fig. 2, this pattern was dominated by a regional decrease extending across premotor, medial frontal, and anterior cingulate regions that shared substantial overlap with the pattern derived in the comparison of HC vs. PD.

### 5.4 Age and gender matched classifiers

**Fig. 3:**
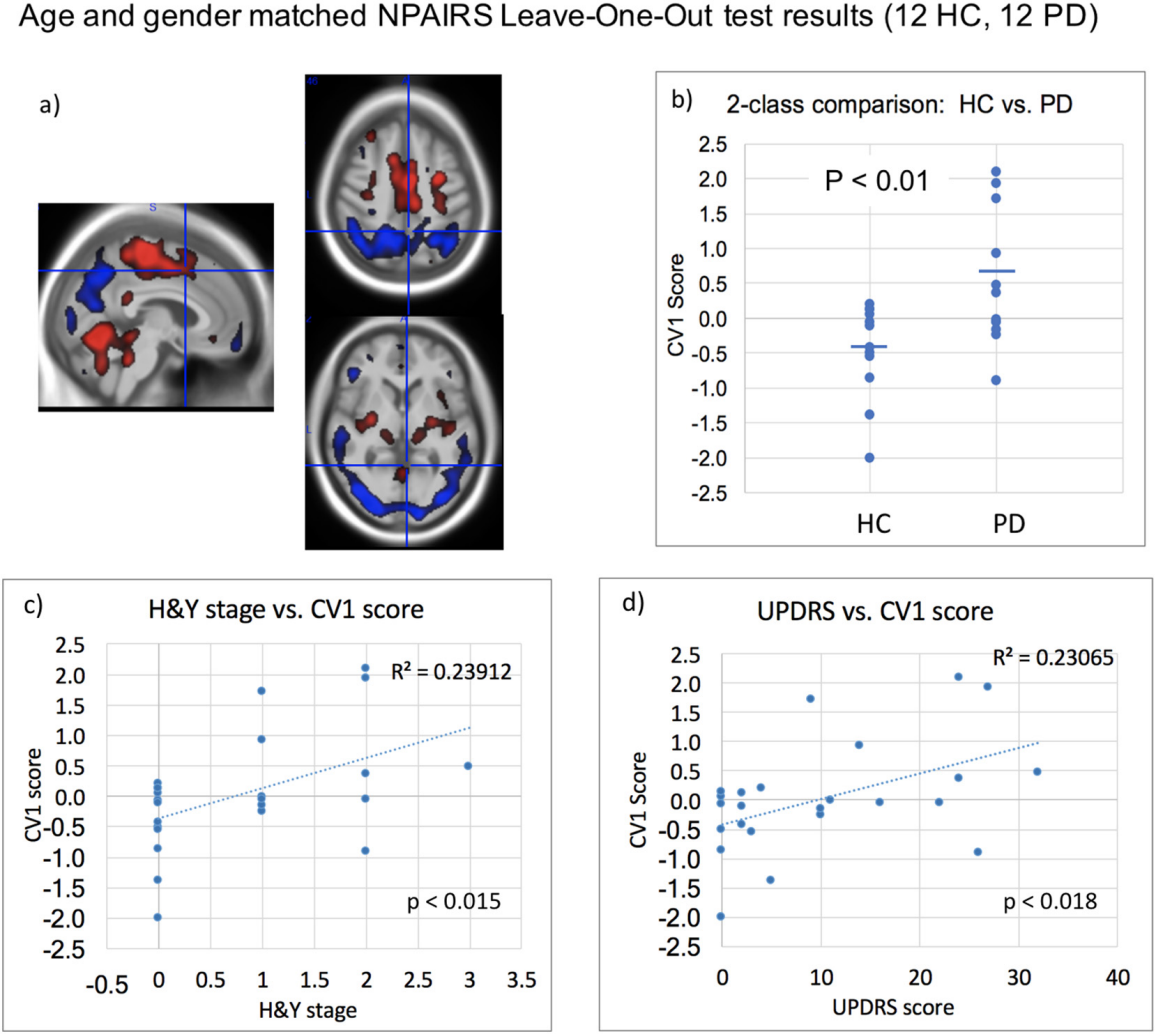
Results of NPAIRS CVA Leave-One-Out test results for comparison of age-matched, gender-matched HC and PD subjects. (a) Pattern of regional hypometabolism (blue) and hypermetabolism (red) relative to whole brain glucose metabolism that discriminated groups; b) CV1 scores; c) Correlation between CV1 score and H&Y stage; and (d) Correlation between CV1 score and UPDRS. higher the CV1 scores reflect greater magnitude of expression of the PDRP relative to whole brain shown in a). Horizontal bars in (b) represent group means; Effect size = −1.11 (Hedge's g). (For interpretation of the references to colour in this figure legend, the reader is referred to the web version of this article.)

The NPAIRS classifier developed using an age- and gender-matched subset of the data (12 HC, 12 PD: 6 H&Y stage I, 5 stage II, 1 stage III) also produced a PDRP like pattern that differentiated HC and PD, shown in Fig. 3. HC and PD group separation was significant (*p* < .010, Effect Size: −1.113, Hedge's g, −1.15, Cohen's d) in Leave-One-Out testing. Compared to the pattern of Fig. 2, the CV1 pattern derived using the age-matched training set showed more occipital cortex hypometabolism in transaxial slices, and less hypometabolism in the premotor, prefrontal, and anterior cingulate structures that dominated the age pattern.

There was no correlation between CV1 score and age in either HC subjects or PD subjects. CV1 scores correlated with H&Y stage (R-squared = 0.24, p < 0.015, Fig. 3c) and with UPDRS scores (R-squared = 0.23, p < 0.018, Fig. 3d).

The SSM age- and gender-matched comparison of HC and PD also produced a pattern that differentiated HC and PD (p < 0.03). As with the NPAIRS analysis, there was no correlation between pattern scores and age, but the SSM scores correlated with H&Y stage (R-squared = 0.20, p < 0.029) and UPDRS (R-squared = 0.18, p < 0.039).

### 5.5 Hoehn & Yahr stage classifier

**Fig. 4:**
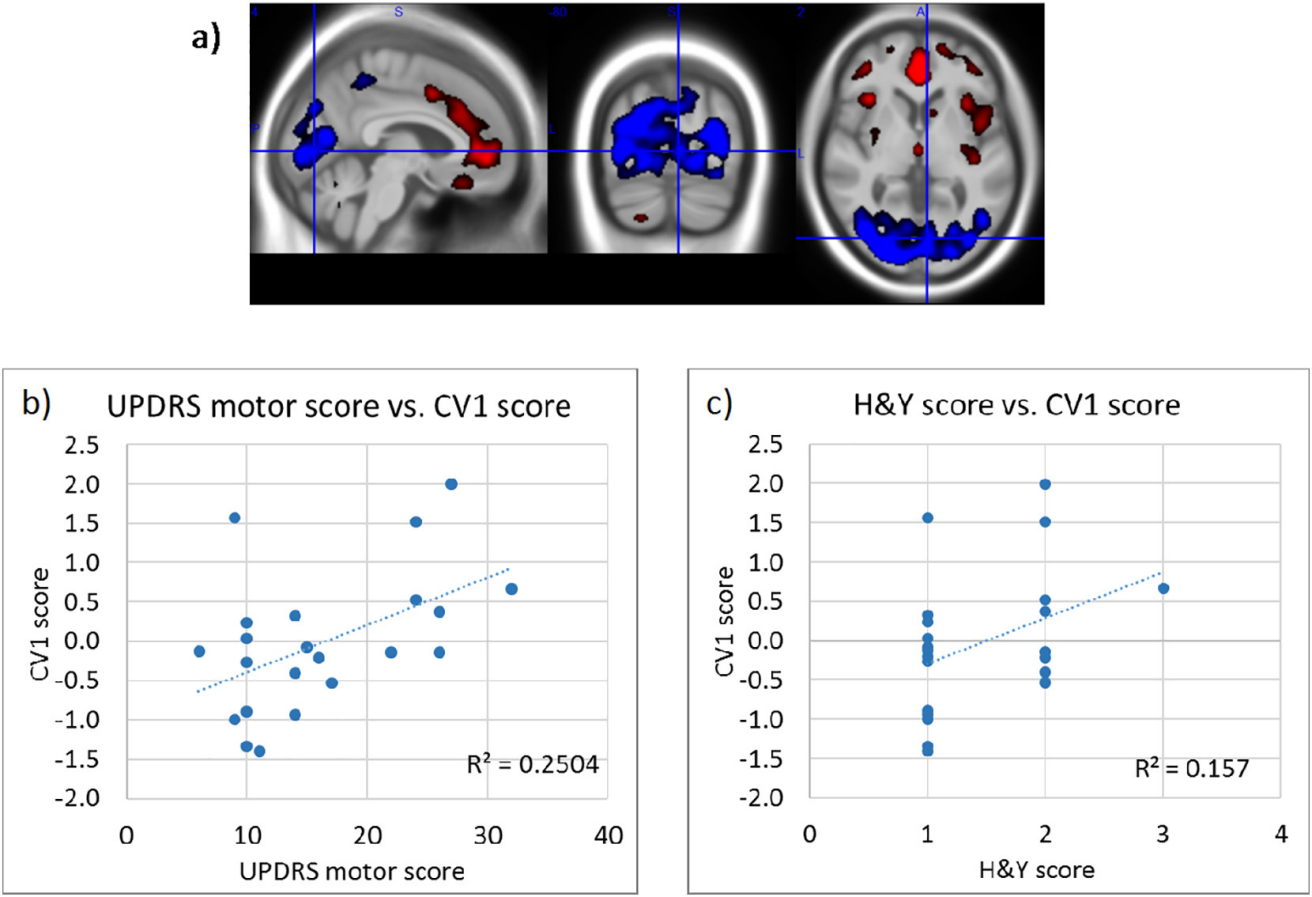
a) CV1 pattern for classifier trained on PD subjects from Hoehn & Yahr stages I vs. II & III; b) Correlation between CV1 scores and UPDRS motor scores generated through Leave-One-Out testing; c) Correlation between Leave-One-Out CV1 scores and H&Y stage. Higher CV1 scores reflect greater magnitude of expression of the PDRP.

The Leave-One-Out results of the NPAIRS CVA analysis of two training classes consisting of PD subjects of H&Y Stage I (N = 13, UPDRS score 11.1 ± 2.8) and stage II & III (N = 9 stage II, 1 stage III, UPDRS score 22.8 ± 5.6), are shown in Fig. 4. The PDRP pattern correlated with UPDRS (R-squared = 0.25, p < 0.015) and H&Y score (R-squared 0.16, p < 0.06). A relationship was observed between CV1 scores and age (R-squared = 0.175), but no correlation remained after removal of one outlier (R-squared = 0.02) (data not shown).

### 5.6 Examination of individual subjects

Inspection of individual subject z-score images showed metabolic asymmetry in only certain H&Y stage I subjects, with the higher classifier scores for stage II more attributable to the magnitude and extent of neurodegeneration as found by Tang et al. (Tang et al., 2010). In particular, z-score images of Stage II patients as compared to Stage I had more extensive occipital hypometabolism, previously shown to correlate with disability in Parkinson's Disease (Bohnen et al., 1999).

## 6 Discussion

Mounting FDG-PET evidence from several unique Parkinson's patient cohorts (published in >15 papers) seems to converge to support a Parkinson's Disease Related Pattern (PDRP) reflecting distinct regional cerebral hypo- and hyper-metabolism in Parkinson's disease relative to whole brain (Eidelberg et al., 1994; Ma et al., 2007; Teune et al., 2013; Wu et al., 2013; Tomše et al., 2017a). However, prior to the present study, the literature lacked confirmation of the PDRP in more than one independent data set not associated with landmark author, and the impact of variation in pre-processing, training models, and model optimization methods was not explored. The findings from the present independent analyses on a unique dataset appear to provide the necessary confirmatory evidence to support the previously published PDRP. The pattern observed in the present study is robust to the variations applied in preprocessing and pattern optimization, as evidenced by the comparison between the SSM and NPAIRS CVA implementations. The key features observed in the present findings are consistent with published studies including hypometabolism in parieto-occipital and premotor/prefrontal cortices and hypermetabolism (or preserved metabolism) in cerebellum, pons, thalamus, lentiform nucleus, and paracentral gyrus, relative to whole brain.

In addition to the independent replication, the present study extends our knowledge of the PDRP by demonstrating evidence of the PDRP in a PD cohort with early disease, with lower overall average H & Y scores (mean H &Y = 1.44 ± 0.58) than previously reported PD cohorts. Notably, previously published studies often derived PDRP patterns using patient cohorts across a broader spectrum of H&Y stages I to V. The fact that a robust pattern could be derived even from among patients with mild illness severity suggests that the PDRP can be detected across all stages of Parkinson's Disease. This finding is consistent with previous studies however with the following noted caveats. Tripathi et al., 2016 observed the PDRP to differentiate PD from Multiple System Atrophy (MSA) and Progressive Supranuclear Palsy (PSP) in similarly mild subjects (mean H&Y in mild subgroup was 1.7 ± 0.5) (Tripathi et al., 2016). Huang et al., 2007 observed longitudinal progression of the PDRP pattern in subjects with a mean H&Y baseline score of 1.2 ± 0.58 (Huang et al., 2007). Direct comparison of these studies to the current study is limited in by the fact that the PDRP had been pre-derived using a different set of subjects with a broader spectrum of disease severity (Ma et al., 2007), whereas the current study demonstrated that the pattern could also be derived in a mild population.

Our analyses further demonstrated the potential for detecting disease progression within the most mild H&Y stages. For example, Leave-One-Out test results from the training of a classifier using subjects from H&Y Stages I and II (one stage III) correlated with UPDRS motor scores and H&Y scores (R-squared = 0.25 and 0.16, respectively; Fig. 4). Since this classifier included only subjects with a diagnosis of PD, the correlations were driven by PD severity rather than by a “binary” difference between HC and PD. The pattern derived using age- and gender-matched subjects also showed correlation of pattern expression with UPDRS scores and H&& scores (R-squared = 0.24 and 0.23, respectively; Fig. 3). However, although pattern scores generally increased with motor severity among the PD patient subset, the correlation significance was driven by the inclusion of both PD and HC subjects. This may have been due to the very limited number of 12 subjects in that subset, and/or to the relative coarseness of the H&Y rating scale. However, it may also suggest that certain pattern features may be more sensitive to disease severity at different stages. From Fig. 4 it can be noted that occipital cortex hypometabolism is prominent, consistent with findings by Bohnen et al. (Bohnen et al., 1999).

Age was identified as a potential confound when deriving the PDRP in the present study, sharing medial frontal aspects of the published PDRP. Removing age bias across groups reduced the medial frontal component while increasing the prominence of the occipital portions of the pattern. Although age is a continuous rather than step-wise factor and could also be modeled using regression approaches, the two group analysis enabled an exploratory extraction of age-driven effects upon metabolism. Even though age-pattern projection into the HC vs. PD classifier was not pursued (due to possible over-compenasation in the present study population), our findings suggest that refinement of the overall disease pattern could include adjustment for age as a continuous variable in studies with larger sample size.

The question of whether the metabolic increases relative to whole brain represent absolute increases or, alternatively, preservation of neuronal activity relative to a declining whole brain has been the topic of multiple studies. Eberling et al. (PD without cognitive impairment) used quantitative PET with arterial blood sampling and found global decreases in metabolic rate with greatest decreases in parietal and visual cortices and thalamus but preservation (rather than an absolute increase) in striatum relative to healthy controls (Eberling et al., 1994). Also using quantitative FDG-PET, Bohnen et al. found absolute meaures of metabolic rate in the thalamus and cerebellum were decreased in PD as compared to HC, but to a lesser extent than parieto-occipital regions (Bohnen et al., 2011). Berti et al. found that when using white matter or other empirically derived reference regions, subcortical region signal intensities were preserved rather than increased (Berti et al., 2012). Cerebral perfusion studies with arterial spin labeled (ASL) MRI, which closely correlates with glucose metabolism (Cha et al., 2013; Verclytte et al., 2015), have found a perfusion pattern similar to the PDRP (Melzer et al., 2011; Le Heron et al., 2014; Teune et al., 2014). Global gray matter perfusion decreased relative to healthy controls and was absent of regional increases if whole brain normalization was not applied (Le Heron et al., 2014). Ma et al. (Ma et al., 2009) found that global metabolic rates were unchanged, aligning with an interpretation of hypermetabolism, and Dhawan et al. showed that the SSM method or other mean-centering approaches do not produce spurious regional increases (Dhawan et al., 2012). In any case, the elevated regions provide a useful combined reference against which the hypometabolic portions of the pattern contrast, making an important contribution to class discrimination and progression measurement. We note that when whole brain normalization or mean centering approaches are applied, regional decreases must be interpreted together with elevation in preserved regions if the overall reference is decreasing.

In multivariate machine learning applications, pre-processing, model selection, and parameter optimization can greatly influence performance. Although two approaches were compared here, class definition, which has major impact upon classifier output, was identical. Both methods also used mean centering and PCA with a discriminant linear model. Yet the consistent features of the PDRP pattern despite differences in specific preprocessing and models, and its regional agreement with univariate studies, suggest that it is robust. We note that for any classifier design and model selection, the choice of re-sampling strategy (such as that employed in NPAIRS), the quality of the input data, and the parameter choices will further impact signal to noise ratio, reproducibility, and prediction, suggested by the slight differences in our analyses.

Limitations in this study included the relatively small sample size and diagnostic constraint to the mild end of the H&Y spectrum. However, the multivariate machine learning approaches employed revealed robust, metabolic patterns in this dataset. Further, the results of this data set are strikingly similar to several larger published data sets from which this data was completely independent. Another limitation was the varied medication status of the subjects. As noted, Parkinson's medications were not taken on the day of the scan. In addition, prior studies have shown that chronically dosed subjects in the “levodopa-on” condition exhibit decreased metabolism in those regions that are elevated (or preserved relative to whole brain) in PD (Berding et al., 2001), which may also arise from mitigation of decreases in other regions. This would have muted the presence of the PDRP-like results in PD subjects, decreasing discrimination between HC and PD, and between H&Y stages. We therefore speculate that a study in medication-naive subjects may likely show discrimination as well. Interpretation of the present findings is also limited by the fact that the PD cohort studied had varied autosomal-dominant and sporadic disease origins.

The tight range of MoCA scores, with mean greater (more normal) than the published mean for MCI subjects (Nasreddine et al., 2005; Hoops et al., 2009; Dalrymple-Alford et al., 2010; Kandiah et al., 2014), precluded identification of a significant cognitive related pattern in this data set. A larger cohort representing a broader cognitive range, may well have permitted the derivation of the cognitive-related pattern. Likewise, the inclusion of additional subjects having a broader spectrum of motor symptoms (e.g. tremor vs. bradykinesia vs. freezing gait) may also have enabled determination of phenotype-specific neurodegenerative patterns.

Since the subjects in the current study did not have amyloid measures, it cannot be confirmed whether certain aspects of posterior cingulate and inferior parietal hypometabolism were associated with comorbid presymptomatic or prodromal Alzheimer's pathology. However, the PD subjects who were age 55 or younger also exhibited these metabolic characteristics, and the probability of AD as a contributor was considered to be low.

In summary, the findings from the present study serve to provide confirmatory evidence that multivariate classification analyses of FDG-PET data can provide a robust, objective, sensitive biomarker of Parkinson's disease. Given this validation, the present findings invite further study of the PDRP. Substantially larger studies could help to identify potential pattern differences associated with disease subtype, and further refine patterns associated with longitudinal disease progression within PD patients. Taken together, these suggest that the Parkinson's Related Disease Pattern has the potential to aid in the measurement of therapeutic effects upon disease progression.

The following are the supplementary data related to this article

Supplemental Fig. 1 Comparison of NPAIRS with Canonical Variates Analysis (CVA) and the Scaled Subprofile Model approach (Eidelberg et al., 1994). Supplemental Fig. 1

## Author’s roles

Dawn Matthews participated in the data analysis and was the primary author on the manuscript. Ana Lukic performed the NPAIRS and SSM analyses and developed the necessary software for implementation. Stephen Strother and Miles Wernick provided scientific direction regarding implementation of the SSM and NPAIRS models. Randolph Andrews performed data quality control and processing. Anat Mirelman provided the imaging and clinical data to ADMdx, and Nir Giladi oversaw its collection. Einat Even-Sapir and Hedva Lerman directed the image acquisition. Jesse Cedarbaum directed the project and its funding. Karleyton Evans provided input and editing to the manuscript.

## Financial disclosures

The analyses in this study were performed by a commercial entity (ADM Diagnostics, Inc.) and funded by Biogen. Dawn Matthews, Ana Lukic, Randolph Andrews, Miles Wernick, and Stephen Strother are employees of ADM Diagnostics, Inc. Jesse Cedarbaum and Karleyton Evans are employees of Biogen. Dr. Giladi serves as consultant to Teva, NeuroDerm, Biogen, Pharma2B, Denali, Abbvie, AccelMed, Monfort and UCB. He receives royalties from LTI and payment for lectures at Teva, UCB, Abbvie, Bial, and the Movement Disorder Society. Prof. Giladi has received research support from the Michael J Fox Foundation, the National Parkinson Foundation, the European Union 7th Framework Program and the Israel Science Foundation as well as from the Teva NNE program, Biogen, LTI, and Pfizer.

## Study funded

Biogen
