# Supplementary material for: FDG PET Parkinson’s disease-related pattern as a biomarker for clinical trials in early stage disease
Source: Neuroimage Clin. 2018 Aug 10;20:572–9. doi: 10.1016/j.nicl.2018.08.006 (PMC6120603; doi:10.1016/j.nicl.2018.08.006)
Supplement: Supplemental Fig. 1 — Comparison of NPAIRS with Canonical Variates Analysis (CVA) and the Scaled Subprofile Model approach (Eidelberg et al., 1994). [file mmc1.docx]

Supplemental Figure 1. Comparison of NPAIRS with Canonical Variates Analysis (CVA) and the Scaled Subprofile Model approach (Eidelberg et al, 1994).
